# Efficiency of attentional processes in attention network theory and autistic symptoms in adolescents with autism spectrum disorder

**DOI:** 10.3389/fpsyt.2022.950245

**Published:** 2022-10-12

**Authors:** Monika Pudło, Ewa Pisula

**Affiliations:** ^1^Department of Psychology, SWPS University of Social Sciences and Humanities, Warsaw, Poland; ^2^Faculty of Psychology, University of Warsaw, Warsaw, Poland

**Keywords:** adolescents, attention, brain networks, executive functions, spatial attention

## Abstract

**Background:**

Attentional impairments in children with Autism Spectrum Disorder (ASD) have been studied extensively, particularly in toddlers and young children. Attentional processes in teenagers with ASD are not fully understood, nor are the relationships between attentional deficits and ASD symptoms in this group.

**Method:**

The aim of this study was to measure the attentional characteristics that attention network theory posits as being related to attention processes: alerting, orientating, and executive attention. We included 37 adolescents (aged 12–20) with ASD and Wechsler IQ in the normal range (≥70) and 37 neurotypical counterparts (NT) matched in terms of age, gender, and IQ. Symptoms of ASD were measured using the Autism Diagnostic Observation Schedule – Second Edition (ADOS-2) and Autism Diagnostic Interview – Revised (ADI-R).

**Results:**

The adolescents with ASD reacted more slowly in all task conditions of the Attention Network Test and committed more errors in six of seven task conditions of this test. There were no group differences in the effects of alerting, orienting, and executive attention. We found moderate correlations of the effect of executive attention with three scales of ADOS-2 (communication, social functioning, and restricted behavior), as well as with the social scale and restricted behavior of ADI-R.

**Conclusion:**

The results indicate that adolescents with ASD performed tasks requiring alerting and orienting attention less efficiently than their counterparts in terms of correctness and reaction time. The relationships between executive attention measures and communication and social affect is discussed.

## Introduction

Autism spectrum disorder (ASD) is a neurodevelopmental disorder that affects social interactions and communication abilities and is associated with restricted interests and activities (1, 2). According to the American Psychiatric Association, the intensity of impairments in these domains varies across the spectrum. Although both clinical observations and scientific studies indicate the prevalence of attentional problems in individuals with ASD, these do not constitute ASD-specific symptoms according to diagnostic classifications (3, 4, 5).

Attention Network Theory (6) divides attention into three domains: alerting, orienting, and executive attention. Each of these domains is underpinned by a specific neural network. Alerting is responsible for maintaining an optimal state of sensitivity to incoming stimuli. Orienting attention helps select and redirect attention to inputs in visual space (6, 7). Executive attention is the central, unitary theoretical construct on which executive function processes depend (8, 9). As defined by Attention Network Theory, executive attention is involved in monitoring behavior and cognitive processes, as well as solving processing conflicts and moderating the activity of sensory, cognitive, and emotional systems (10). Following the terminology used in Attention Network Theory, executive attention is responsible for volitional control of cognitive processes and maintaining a state of vigilance (11). In other neurocognitive theories, attentional processes controlling volitional performance of goal-oriented tasks are referred to as top-down control or executive functions (8).

Keehn, Lincoln, and Mueller (12), Fan et al. (13), and Mutreja, Craig, and O’Boyle (14) studied attention processes in individuals with ASD with the use of the computerized Attention Network Test (ANT) for a detailed description of the ANT, see the Methods section) (14). Applied an adjusted version of ANT (15) (Rueda et al. (15) in children aged 5–11 years with ASD and their neurotypical counterparts. They did not find differences between groups in alerting, orienting, or executive attention. However, children with ASD reacted more slowly in tasks with cue conditions for orienting and in tasks with flanker conditions for executive attention. The study had a relatively small group of children with ASD (*N* = 14), which constitutes a severe limitation. The study of Keehn et al. (12) used ANT in adolescents (aged 8–19 years) with ASD. The neurotypical (NT) group was matched to the ASD group in terms of IQ and age (13). There were no differences between the groups in alerting and executive attention; however, orienting was worse in the ASD group. The adolescents with ASD reacted more slowly in flanker tasks (which is regarded as an indicator of executive attention) and in tasks with cue conditions that measured orienting. A relationship between the intensity of attentional performance and the symptoms of ASD was also observed. The study also showed a significant correlation between alerting and social affect. However, the results of another study conducted with a group of boys with ASD aged 10–15 years showed that they did not differ from their typically-developing counterparts nor from boys with ADHD in the number of correct responses on ANT tasks or reaction time for alerting, orienting, and executive attention (16).

In a study by Fan et al. (13), who developed the ANT, individuals with ASD made a higher number of errors on ANT tasks involving alerting and executive attention than did the control group (both groups had 12 participants; mean age = 30 years). No differences between the study groups were found in reaction time on tasks measuring alerting, executive attention, and effects of networks.

The first aim of this study was to investigate levels of alerting, orienting, and executive attention in adolescents with ASD with a normal range of intelligence, and to compare the results to NT group. Most studies on attention in individuals with ASD focus on toddlers and infants, whereas attentional processes in adolescents are still underexplored. Based on the results from previous studies conducted on children and teenagers with ASD, described above (4, 14, 16), it could be inferred that alerting and orienting in adolescents with ASD and a normal range of intelligence may differ from that of their typically developing counterparts because of the biological underpinnings of these processes. Such differences emerge at an early age and are robust to therapy, in contrast to executive attention processes, which continue to mature until adulthood and can be compensated for. Furthermore, a review of 37 papers about visual disengagement processes and spatial orienting tasks in individuals with ASD, which is controlled by the orienting network, indicate that deficits in visual disengagement continue into adulthood (17). We did not expect group differences in indicators of executive attention processes, as most adolescents with ASD within the normal range of intelligence can communicate fluently (18), and fluent communication abilities and language knowledge enable them to use inner speech, which mediates some executive processes (e.g., task switching and flexibility) (19). So, based on the results of previous studies, we formed the following hypotheses: (1) alerting and orienting in adolescents with ASD will be lower than in their NT counterparts, and (2) individuals with ASD will not differ from their NT counterparts in executive attention.

The second aim of the study was to verify whether alerting, orienting, and executive attention are linked with intensity of autistic symptoms in adolescents with ASD. The attentional characteristics are not included in the diagnostic symptoms criteria. However, several studies have investigated the relationship between attention and social interaction impairment (for example (20, 21) and stereotyped behavior (22, 23) in infants with ASD. The results do not indicate that attention difficulties can be directly connected with autistic symptoms. So since there are insufficient theoretical grounds and premises from the results of previous studies, we do not propose any hypotheses regarding the relationship between attention processes and the severity of ASD symptoms.

## Materials and methods

### Participants

The inclusion criteria for the ASD group were as follows: IQ in the normal range (full scale Wechsler IQ ≥ = 70); a psychiatric diagnosis of childhood autism or Asperger’s syndrome based on the International Classification of Diseases, 10th revision (ICD-10) criteria (WHO, 1992); and being aged between 12 and 20 years. For the purposes of the study, only participants who reached the cutoff scores for a diagnosis of ASD on both the Autism Diagnostic Observation Schedule-2 (ADOS-2) (24), in Polish adaptation by Chojnicka and Pisula (25) and the Autism Diagnostic Interview-Revised (ADI-R) (26) in Polish adaptation by Chojnicka and Pisula were included (27).

In order to be able to employ the computerized attention test, we used the following inclusion criteria: lack of neurological comorbidities (excluding ADHD diagnosis) and absence of attention-stimulating medication. One participant had ADHD. However, he had never received medication for it. We decided to include him in the group, because he met all inclusion criteria regarding ADOS-2 and ADI-R cut-offs, IQ level, and lack of medication. Participants on medications were not included in the study. Adolescents with ASD were recruited through diagnostic and psychiatric centers as well as schools in Poland. Initially, this project included 51 adolescents with a diagnosis of ASD and 50 typically developing counterparts in the control group.

Ultimately, these criteria were satisfied by 37 of the recruited individuals with ASD (35 boys and 2 girls); therefore 37 controls, who were well matched in terms of age, gender, and Full Scale IQ to the teenagers with ASD were included into study. Autism spectrum disorder is about 4–5 times more common among males than among females (28), hence the disproportionately large number of male participants in the study. Because we set such rigorous criteria for diagnosis, lack of medication, and neurological comorbidities, we were not able to recruit more girls into the study.

Information on the participants’ age, sex, and intellectual ability, as well as ADOS-2 and ADI-R scores, are given in Table 1.

**TABLE 1:** Characteristics of ASD group and control group (IQ, ADOS, ADI-R).

				IQ		ADOS-2 Scales			ADI-R Scales		
							
Subjects	Group	Age (yrs)	Verbal	Non-verbal	Full-scale	Social	Communication	Repetitive behavior	Social	Communication	Repetetive behaviour
A1	ASD	12.17	102	72	90	7	5	2	24	13	9
A2	ASD	13.08	105	100	103	5	8	5	13	20	7
A3	ASD	12.5	122	151	139	20	16	4	18	25	7
A4	ASD	13.5	144	137	144	4	4	2	23	19	10
A5	ASD	14.67	110	102	107	11	8	1	25	20	12
A6	ASD	15	87	117	102	9	7	2	13	13	5
A7	ASD	14.5	92	95	93	7	3	5	16	18	11
A8	ASD	16.08	85	86	85	15	5	1	12	18	10
A9	ASD	15.5	126	118	124	17	6	2	20	17	9
A10	ASD	18.58	103	100	102	17	14	6	14	21	10
A11	ASD	18.75	113	109	112	9	3	5	23	19	10
A12	ASD	17.75	121	100	112	13	12	7	12	12	8
A13	ASD	19	82	90	85	8	8	2	21	18	6
A14	ASD	17.75	84	74	79	12	7	2	26	12	6
A15	ASD	16.33	147	100	127	5	3	5	23	17	12
A16	ASD	17.5	96	69	84	11	6	2	3	16	8
A17	ASD	15.25	96	128	113	11	10	0	21	12	3
A18	ASD	18.42	99	73	87	12	6	0	18	9	5
A19	ASD	12.08	106	80	94	7	6	3	26	22	9
A20	ASD	12.58	99	107	103	7	10	1	23	18	8
A21	ASD	13	59	107	81	10	7	3	20	18	11
A22	ASD	13.42	122	123	124	15	7	4	29	25	15
A23	ASD	12.75	91	87	88	8	6	3	7	10	3
A24	ASD	14.17	82	93	86	11	7	1	26	25	5
A25	ASD	15.08	90	90	89	25	18	3	13	13	6
A26	ASD	20.25	98	96	97	16	12	3	26	18	6
A27	ASD	18.5	78	77	80	8	7	4	7	24	9
A28	ASD	19.58	99	107	102	8	4	0	24	16	10
A29	ASD	20.08	120	120	114	6	4	4	18	7	7
A30	ASD	16.67	90	102	95	9	4	4	21	14	5
A31	ASD	13.92	79	79	77	6	4	3	19	19	11
A3	ASD	12.58	123	133	130	7	4	6	28	18	10
A33	ASD	12.37	96	106	101	10	8	7	28	23	6
A34	ASD	14.92	74	90	80	14	7	3	19	19	8
A35	ASD	13.67	128	118	126	5	6	2	25	25	11
A36	ASD	15.33	117	114	117	7	5	1	23	19	12
A37	ASD	16.42	105	114	110	10	6	1	26	19	8
Mean		15.50	101.89	101.73	102.22	10.32	7.11	2.95	21.40	18.31	8.28
(s.d.)		2.50	19.33	19.39	17.90	4.59	3.51	1.90	4.88	4.17	2.74
C1	Control	16.33	79	95	86						
C2	Control	17.33	78	78	76						
C3	Control	12.58	108	133	122						
C4	Control	13.83	96	107	102						
C5	Control	18.33	99	96	98						
C6	Control	19.83	100	92	97						
C7	Control	13.08	104	93	99						
C8	Control	13.42	104	100	102						
C9	Control	13.58	93	82	87						
C10	Control	13.58	96	108	102						
C11	Control	12.67	118	124	123						
C12	Control	16	78	103	88						
C13	Control	18.83	109	99	105						
C14	Control	18.75	108	107	108						
C15	Control	17.92	94	97	95						
C16	Control	19.33	121	77	105						
C17	Control	17.17	126	118	123						
C18	Control	19.58	100	89	105						
C19	Control	18.83	123	113	119						
C20	Control	16.25	99	108	99						
C21	Control	18.5	122	110	117						
C22	Control	14.58	92	103	97						
C23	Control	12.17	142	102	125						
C24	Control	14.67	111	83	98						
C25	Control	12.83	109	92	101						
C26	Control	13.92	140	100	123						
C27	Control	12.08	150	128	144						
C28^#^	Control	17.42	100	101	104						
C29	Control	15	88	109	98						
C30	Control	17.17	112	109	111						
C31	Control	16.25	116	118	117						
C32	Control	13.5	113	114	115						
C33	Control	14.67	95	99	96						
C34	Control	14.5	116	130	124						
C35^#^	Control	15.5	120	107	116						
C36	Control	16.33	103	101	102						
C37	Control	18.42	81	67	75						
mean		15.80	106.57	102.49	105.51						
s.d.		2.37	17.095	14.681	14.510						

ADOS-2 module 3 cut off autism spectrum diagnosis = 7; ADOS-2 module 4 cut off ASD diagnosis = 6.

^#^Girls ASD group n = 2, Control group n = 2.

RT- reaction time.

We used the Social Communication Questionnaire (SCQ) (29); Polish adaptation by Pisulaet al. (30) and Autism Quotient (AQ) (31), Polish version by Pisula et al. (32) to exclude from the NT group individuals who would obtain cutoff scores for symptoms of autism spectrum disorder. The NT group was recruited through schools.

The study was approved by the Ethics Committee of the Faculty of Psychology at the University of Warsaw, Poland.

### Measures

*The Autism Diagnostic Observation Schedule-2 (ADOS-2)* is a semi-structured observation protocol used to assess autism-related social and communication behavior (24). Participants under the age of 15 were administered Module 3 of ADOS-2. Assessment of the behavior of adolescents aged 16 and older was based on Module 4 of ADOS-2. We used the cut-off points of the Polish version of ADOS-2 (25). Modules 3 and 4 of the Polish validation of ADOS-2 showed high reliability. The Cronbach’s alpha value for the Social Affect (SA) scale for Module 3 was 0.90, while for Module 4 it was 0.92. The alpha value for the Restricted/Repetitive/Stereotyped Interests/Behaviors and Activities scale was 0.68 for Module 3 and 0.76 for Module 4.

*The Autism Diagnostic Interview-Revised (ADI-R)* is a semi-structured interview for use by the parents or caregivers of a person with ASD (26) Polish adaptation by Chojnicka and Pisula, (27, 29). It consists of 111 items across three scales: Communication, Social Development, and Repetitive, Restricted, and Stereotyped Behavior. Cronbach’s alphas were 0.85–95 for Social Affect and Communication. The alpha value for Restricted, Repetitive Behavior was 0.63 and for Reciprocal Peer Interactions (RPI) was 0.64.

*Autism Quotient* (AQ). This questionnaire consists of 50 statements referring to five domains of autism: Social Skills, Attention to Detail, Attention Switching, Communication, and Imagination (31). For teenagers aged from 12 to 15.11 years old, the parents fill in the questionnaire. The self-administered version of AQ is designed for adolescents above 16 years of age. The Cronbach’s alpha value of the adapted Polish self-administered AQ was 0.86 for individuals with ASD and 0.75 for NT individuals (32).

*The Social Communication Questionnaire (SCQ)* is a questionnaire that consists of 40 “yes or no” items to be completed by parents in order to screen for the presence of ASD. Cronbach’s α in the Polish adaptation of the tool was 0.92 for the overall score (30).

*Wechsler Intelligence Scale.* Depending on the age of the participants, IQ was measured using either the Wechsler Intelligence Scale for Children (33) or the Wechsler Adult Intelligence Scale (WAIS-R) (34). The result of the Digit Span subtest operationalizes the executive function process of working memory. The Cronbach’s alphas were 0.73–0.96 for subscales of the Wechsler Intelligence Scale for Children and 0.76–0.87 (33). The Cronbach’s alphas for the Wechsler Intelligence Adults Scale were moderate to high (0.52–0.90), whereas subscales were characterized by high alphas (0.8–0.9) (34).

**FIGURE 1:**
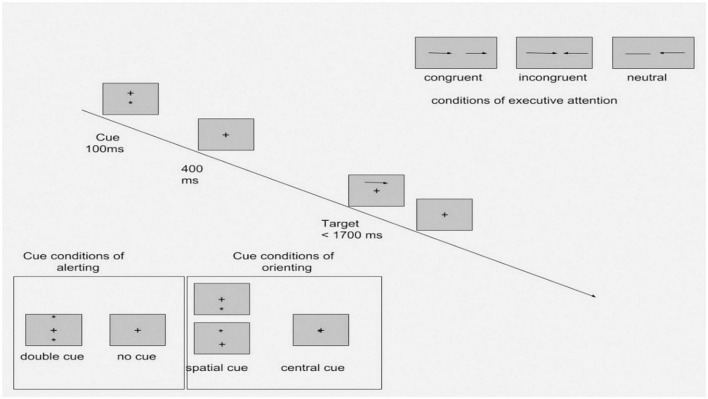
Experimental procedure of the ANT with four cue conditions and three flanker conditions (Adapted from Fan et al. (46).

*The Attention Network Test (ANT)* is a set of computerized tasks designed to measure the efficacy of three attention networks – alerting, orienting, and executive function – based on attention network theory. The design of the ANT is based on the original version of attention network theory (35). The ANT consists of seven cue conditions: no cue, double cue, spatial cue, center cue, and flanker (congruent, incongruent, and neural). The double cue and no cue conditions test alerting efficacy. Orienting efficacy is measured in the spatial cue and center cue conditions. The flanker conditions assess the capacity of the executive function responsible for resolving cognitive conflicts. The efficacy of alerting was calculated by subtracting the mean reaction time (RT) of the double cue condition from the mean RT of trials with no cue conditions. The orienting effect was calculated by subtracting the mean RT of trials with spatial cue conditions from the mean RT of the center cue condition. Efficacy of executive attention was calculated by subtracting the mean RT of trials with incongruent flanker conditions from the mean RT of congruent conditions. Any RTs shorter than 100 ms or longer than 1,200 ms were excluded from the calculation of RTs. The test–retest mean RTs for seven ANT task conditions were highly correlated between two sessions: *r* = 0.87. The reliability of alerting, orienting, and executive attention ranged from 0.52 to 0.77 (35). The design of the ANT is presented in Figure 1.

The processes and the measures of ANT are presented in Table 2.

**TABLE 2:** Attentional processes and their measures.

Attentional measure	Tests
Alerting	*Attention Network Test (ANT)* - The number of correct responses and reaction time for no cue condition - The number of correct responses and reaction time for double condition - The alerting effect: subtracting mean RT of the double cue conditions from the mean RT of the no cue conditions.
Orienting	*Attention Network Test (ANT)* - The number of correct responses and reaction time for center cue - The number of correct responses and reaction time for spatial cue - The orienting effect: subtracting mean RT of the double cue conditions from the mean RT of the no cue conditions.
Executive Attention	*Attention Network Test (ANT)* - Solving conflict: the number of correct responses and reaction time for three flanker conditions: congruent, incongruent, neutral - The effect of executive attention: subtracting the mean RT of all congruent flanking conditions from all incongruent flanking conditions

RT- reaction time.

### Data analysis

Analyses were performed using IBM SPSS Statistics 22.0. Responses were filtered if the execution time was shorter than 100 ms or longer than 1,260 ms in the control group, and shorter than 100 ms or longer than 1,500 ms in the ASD group, based on an upper and lower cutoff of 2.5% of observations (similar criteria for filtering responses in ANT were applied by Tversky and Kahneman (36), in which trials below 200 ms and above 1,500 ms were excluded).

The procedure for filtering responses/observations was as follows.

The full sample consisted of 101 participants: 51 with ASD and 50 from the control group. We excluded RTs shorter than 100 ms, as this indicates a random response (37). Excluding responses shorter than 100 ms from our data was equivalent to excluding 2.5% of the shortest RT responses. Given that long responses can also be considered random or outliers (37),

We decided to apply the same criteria for the longest RTs, with a cut-off at 2.5% of the longest responses.

The same 2.5% cut-off criterion was applied to both ASD and control groups. For the data collected with the ASD group, the 2.5% threshold was 1,500 ms and we excluded 233 measurements. In the control group, the 2.5% threshold for the highest was 1,260 ms; a total of 211 measurements were excluded.

After filtering the measurements, we also excluded 14 participants with ASD due to their diagnosis not having been confirmed by ADOS-2 nor ADI-R scores. Our aim was to keep the ASD sample homogenous in terms of clinical symptoms. As a result, the sample with individuals with ASD consisted of 37 adolescents. We matched the control group in terms of full scale Wechsler IQ and age to every individual with ASD kept in our analysis, as we wanted to avoid demographic and cognitive differences between the clinical and ASD groups. Ultimately, our analysis included 74 participants: 37 in the ASD group and 37 in the control group.

Since most of the variables either showed a homogeneous variance or normal distribution (see the results of Kolmogorov–Smirnov test in Supplementary material), we used the parametric analysis of variance (ANOVA). All reported *p* values were adjusted with Bonferroni corrections for multiple comparisons.

### Procedure

All research sessions were conducted either in the research session room of the University of Warsaw Psychology Department or in a room at a medical center in which the adolescents had participated in therapy and/or training sessions.

Participants completed the Attention Network Test on a laptop computer with a 15.6-inch monitor with the use of E-Prime software. The experiment consisted of 144 trials.

The number of trials was lower than in the original version (35). We decided to create a shorter test, because in contrast to (35), who examined neurotypical adults, we were examining adolescents with a neurodevelopmental disorder. Before undergoing the test, each participant completed three training blocks. Each experimental session with ANT lasted about 15–20 min depending on the length of the participant’s break between experimental sessions.

The session with ADOS-2 took place after a short break following the administration of the ANT. The ADI-R was conducted with the parents of the adolescents with ASD over a period of roughly 90–120 min, either after the test session with the participant or on another day.

The Wechsler Scale results of 18 participants were provided by medical centers with the written agreement of the participants’ parents. If WISC-R or WAIS-R results from the preceding 12 months were not available, the participants and their parents were asked to schedule the test for a day different from the administration of ANT and ADOS-2, but not later than 1 month after the first test session.

## Results

### Differences in attentional performance between autism spectrum disorder and neurotypical groups

Table 3 presents descriptive statistics from the ANT for the ASD and control groups.

**TABLE 3:** Descriptive statistics of attentional tests measures in ASD Group and Control Group.

Attention Network Test (ANT)	ASD Group (*N* = 37) M(SD)	Control Group (*N* = 37)
Alerting	−3.89 (75.32)	20.91 (36.85)
RT no cue condition	845.33 (186.37)	654.21 (118.69)
RT with double cue	845.33 (186.37)	654.21 (118.69)
Correct responses with no cue	24.78 (8.09)	32.16 (5.69)
Correct responses with double cue	25.84 (7.42)	31.86 (5.85)
Orienting	36.21 (58.99)	52.02(43.62)
RT center cue	849.23 (195.63)	633.29 (115.09)
RT spatial cue	813.02 (191.86)	581.27 (115.53)
Correct responses with center cue	25.78 (7.99)	32.08 (5.91)
Correct responses with spatial cue	25.97 (8.15)	32.49 (6.20)
Executive attention	102.04 (102.87)	139.52 (86.93)
RT congruent flanker	818.02(192.26)	607.46 (127.66)
RT incongruent flanker	29.54 (13.78)	746.97 (172.24)
RT neutral flanker	778.39 (188.31)	557.10 (113.053)
Correct responses congruent flanker	36.35 (10.81)	46.13 (4.88)
Correct responses incongruent flanker	29.54 13.78)	36.03 (17.57)
Correct responses neutral flanker	36.48 (10.97)	46.46 (3.06)

RT-reaction time. Figure 1. Experimental procedure of the ANT with four cue conditions and three flanker conditions (Adapted from Fan et al. (46).

**FIGURE 2:**
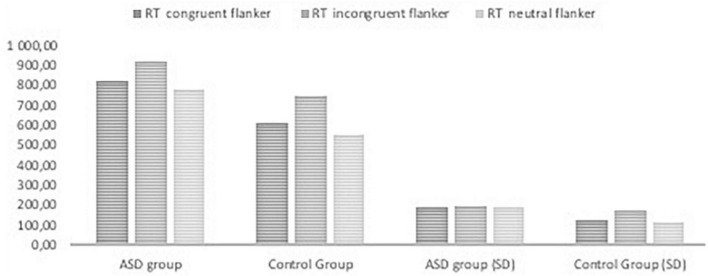
Reaction time in the ANT tasks with congruent, neutral, and incongruent flankers in ASD group and control group.

**FIGURE 3:**
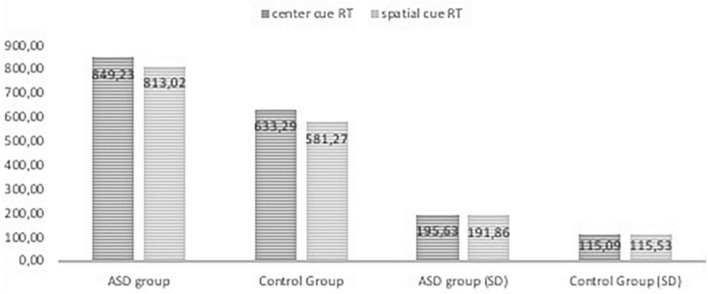
Reaction time in the ANT tasks with center cue and spatial cue conditions in ASD group and control group.

**FIGURE 4:**
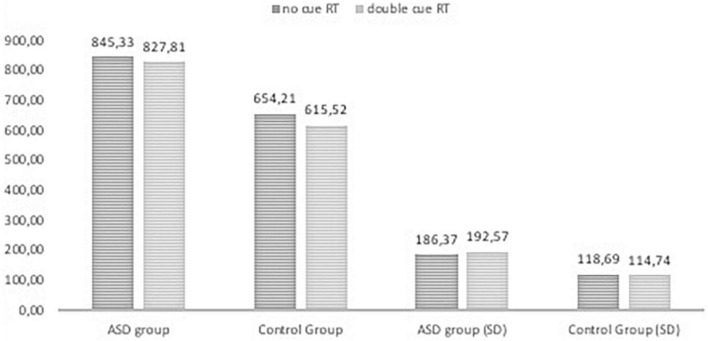
Reaction time in the ANT tasks with nor cue and doublel cue conditions in ASD group and control group.

Figures 2, 3, 4 Mean of reaction time in ASD and in control group.

The results of RTs in the following seven conditions include two persons who made many mistakes; these persons were excluded from analysis of attentional effects. The repeated measures ANOVA analysis revealed a significant main effect of interaction group with reaction time in seven conditions, F _(2.976, 214,243)_ = 3.513, p < 0.005, η _p_^2^ = 0.047; Greenhouse-Geisser corrected.

The RTs in all task conditions were significantly longer in subjects with ASD than in the NT group: double cue, F_(1,71)_ = 33.18, p < 0.001, η^2^ = 0.315; no cue, F_(1,72)_ = 27.683, p < 0.001, η^2^ = 0.278; spatial cue, F_(1,72)_ = 39.61, p < 0.001, η^2^ = 0.355; center cue, F_(1,72)_ = 33.49, p < 0.001, η^2^ = 0.317; congruent flanker, F_(1,72)_ = 30.79, p < 0.001; η^2^ = 0.300; incongruent flanker, F_(1,72)_ = 15.897, p < 0.001, η^2^ = 0.181; and neutral flanker, F_(1,72.)_ = 37.55, p < 0.001; η^2^ = 0.343.

There was no interaction effect group with the correct number responses across seven conditions in ANT, F_(1.278,92,001)_ = 2.074 p = 0.148, η _p_^2^ = 0.028; Greenhouse-Geisser corrected.

The differences in attentional effects between groups were not significant: alerting, F_(1,72)_ = 3.239, p = 0.076; orienting, F_(1,72)_ = 1.717, p = 0.194; and executive attention, F_(1,72)_ = 2.865, p = 0.095.

### Correlation analysis autism diagnostic observation schedule – second edition and attention network test

A correlation analysis was performed on ADOS-2 scales and ADI-R scales (social affect, communication, stereotyped behavior), AQ scales and the seven ANT measures. Only correlations r > 0.4 at a significance of p < 0.01 are reported. The correlation coefficient above 0.4 is considered as moderate according to Guilford classification. Because of small clinical sample the smaller correlation could be random (38). We included into Supplementary materials the table with correlations. However, only two low correlations of the number of correct responses were revealed in incongruent conditions: with AQ Social skills scale (r = −0.398; p < 0.01) and with AQ scale Attention to details. (r = −0.330; p < 0.05).

There was only one moderate correlation between executive attention and the communication scale from ADOS-2 (r = −0.476; p < 0.01). Additionally, the effect of executive attention was correlated with the restricted behavior (r = 0.429^**^; p < 0.01). There was also the correlation of ADI-R social scale (r = −0.536; p < 0.01), however, after Sidak correction for the significant alpha level, it was insignificant.

## Discussion

In comparison with the neurotypical group adolescents with ASD made more mistakes and reacted more slowly in the seven ANT task conditions. There were no significant differences between the groups in alerting, orienting, or executive attention, nor were there differences in perceptual search and switching. The studied groups were also compared in terms of executive attention indicators measured with the ANT. Executive attention was examined in tasks with three stimulus/distractor variants (congruent, incongruent, neutral), which indicate ability to resolve a cognitive conflict (35), but no differences were found.

The results of other studies using ANT also did not indicate any differences in executive attention between NT and ASD groups (12, 16). A study by Fan et al. (13), in which a modified version of the ANT was used to identify areas of the brain responsible for attention processes, showed that adults with ASD performed worse on tasks measuring executive attention and alertness than do typically developing individuals, matched for age and level of mental development. In the study presented in this paper, adolescents with ASD made more errors in tasks measuring executive attention than did their NT counterparts. However, there were no differences between the groups with regard to reaction time on the incongruent flanker task, which requires executive attention processes such as inhibition, planning, and working memory at the same time. On the other hand, the ASD group in our study reacted more slowly when performing the congruent flanker and neutral flanker tasks. This might be due to their enhanced perceptual performance capacity, which enables proper reaction without the involvement of executive attention (39). That is to say – when exposed to the stimuli, individuals with ASD processed visual details more quickly than their NT counterparts. However, this is a hypothetical explanation which could not be proven in this study.

The results of this study suggest the following conclusions about attention processes in adolescents with ASD with normal IQ: the efficiency of attention processes triggered by external stimuli was lower in the areas of alerting and orienting. Executive attention in this group was also less efficient than in their typically developing counterparts in terms of reaction time and correctness. In the task with the incongruent flanker, which also measures executive attention, individuals with ASD did not differ from their NT counterparts in terms of correctness.

Similar results were obtained by Keehn et al. (12). In their study, the ASD group differed from the NT group in terms of correctness, but not in RTs, as they did in our study. The task with incongruent flankers requires “problem solving” (35). Alongside executive functions, processes such as updating, inhibition, and switching play important roles in attention and the successful completion of such tasks (9). The results of our study and that of Keehn et al. (12) support the finding that individuals with ASD exhibit difficulties in tasks that demand coordination of a variety of processes at the same time. The results of our study are similar to (12, 14), however the range of age is different. We studied the group on the whole range of adolscance from 12 till 20 years old, wheareas in the Mutreja (14) study there were children between 5 and 11 years old and the group was larger (our study ASD n = 37, in (14) ASDn = 14 in (12) ASD n = 20).

This was the case in a study involving the use of the dual tasks, in which individuals with ASD also performed less efficiently than their NT counterparts (40). In consequence, individuals with ASD react either more slowly or less correctly than do their NT counterparts in tasks with incongruent flankers. One metanalysis of 18 research papers on visual orienting in autism showed that participants with ASD were most impaired in arrow cue tasks (41). This is additional evidence that more detailed and more complex tasks that rely on ‘problem solving’ are more difficult for participants with ASD. The slow reaction in individuals might be caused also by the motor impairments, which is reported in the individuals with ASD (42). Hence they pressed the buttons on the keyboard. In addition the results from Diffusion Tensor Imaging (DTI) and task-related functional imaging study indicate on the weak cortico-cerebellar connectivity in individuals with ASD (43). This disruption can cause delay in the reaction time in all cognitive task (44). However in our study we did not control motor skills of participants, so this conclusion is hypothetical. In subsequent studies on attention in individuals with ASD, it would be worth including the tests for motor skills. A relationship between attention and severity of autistic symptoms was found only for the effect of executive attention and communication. The correlation was negative, meaning that if the difference between congruent and incongruent flanker tasks was small, the result of Communication on ADOS-2 was high. Due to the fact that interpretations of effects on the Attention Network Test have been criticized (12, 16) it cannot be concluded that low executive attention efficiency is linked with high Communication on ADOS-2. In the study by Keehn et al. (12), a correlation between alerting and social communication was found. However, the age range of participants differed between the study of Keehn et al. (10–15 years) and our study (12–20 years). The relationship between symptoms and attention might be less remarkable in older individuals with ASD because of the development of voluntary control. As a result, voluntary control and motivation can compensate for deficits in orienting attention and alerting processes (45). We did not perform regression analysis because we assume that the attentional performance and the severity of autistic symptoms are interdependent in adolescents. Perhaps in infants with ASD, the research on the direction, if attention is primary to autistic symptoms severity, would be helpful for diagnosis and therapeutic intervention.

## Limitations and implications

In contrast to previous studies using ANT with individuals with ASD, our study found a relationship between attentional performance and intensity of symptoms reported by parents of adolescents with ASD in the standardized ADI-R interview and measured with the ADOS-2 standardized observational protocol. We obtained a moderate correlation of executive attention with social skills and restricted behavior. This relationship could mean that goal-directed behaviors play an important role in social interactions and during stereotyped behaviors.

This study has some limitations. It was conducted on a group of individuals aged 12 to 20 years, representing the wide range of adolescence. A longitudinal study would be more valuable as it could capture the development of attention functions and changes in the efficiency thereof. It would also be interesting to apply qualitative methods to attention functioning, such as the BRIEF (42), or interviews with teachers. This would allow us to examine whether the results of psychological tests are congruent with the daily functioning of study participants.

## Data availability statement

The original contributions presented in this study are included in the article/Supplementary material, further inquiries can be directed to the corresponding author/s.

## Ethics statement

The studies involving human participants were reviewed and approved by Ethics Committee of the Department of Psychology of University of Warsaw. Written informed consent to participate in this study was provided by the participants’ legal guardian/next of kin.

## Author contributions

All authors listed have made a substantial, direct, and intellectual contribution to the work, and approved it for publication.
