# Supplementary material for: Efficiency of attentional processes in attention network theory and autistic symptoms in adolescents with autism spectrum disorder
Source: Front Psychiatry. 2022 Oct 12;13:950245. doi: 10.3389/fpsyt.2022.950245 (PMC9614655; doi:10.3389/fpsyt.2022.950245)
Supplement: Supplementary file 2 [file Table_1.DOCX]

Supplemantory Table

Explanation of labels in database and in the table of correlations.

| **Label** | **Explanation** |
| --- | --- |
| Subject_code | The anonymized code of the subject |
| Group_ASD1_Control2 | ASD- autism spectrum disorder, control- participant from control group (1-ASD; 2-Control) |
| age_years | Age in years |
| age_months | Age in months |
| age_numerical_variable | Age in years and months, calculated for analyses |
| Alerting_ANT | The alerting effect in Attention Network Test |
| Orienting_ANT | The orienting effect in Attention Network Test |
| E_ANTxecutive_att_ANT | The executive attention effect in Attention Network Test |
| N_Correct_Practice_Congruent_ANT | Correct responses congruent flanker in Attention Network Test |
| N_Correct_Practice_neutral_ANT | Correct responses neutral flanker in Attention Network Test |
| N_Correct_Practice_incongruent_ANT | Correct responses incongruent flanker in Attention Network Test |
| RT_congruent_ANT | Reaction time congruent flanker in Attention Network Test |
| RT_neutral_ANT | Reaction time neutral flanker in Attention Network Test |
| RT_incongruent_ANT | Reaction Time incongruent flanker in Attention Network Test |
| N_Correct_Practice_centercue_ANT | Correct responses with center cue in Attention Network Test |
| N_Correct_Practice_no_ANT | Correct responses with no cue in Attention Network Test |
| N_Correct_Practice_spatial_ANT | Correct responses with spatial cue in Attention Network Test |
| N_Correct_Practice_double_ANT | Correct responses with double cue in Attention Network Test |
| RT_center cue_ANT | Reaction time center cue in Attention Network Test |
| RT_no_cue_ANT | Reaction time no cue in Attention Network Test |
| RT_spatial_cue_ANT | Reaction time spatial cue |
| RT_double_cue_ANT | Reaction time double cue |
| IQ_full_ scale | Intelligence quotient full scale |
| IQ_Verbal | Intelligence quotient in verbal scale |
| IQ_Non_Verbal | Intelligence quotient in non- verbal scale |
| AQ_total_scores | Total scores in Autism Quotient |
| AQ_SocialSkill | The social skills scale in Autism Quotient |
| AQ_Att_switch | scale in Autism Quotient |
| AQ_Att_details | scale in Autism Quotient |
| AQ_Communication | scale in Autism Quotient |
| AQ_Imagination | scale in Autism Quotient |
| AQ_cut_off | Cut off scores in Autism Quotient (1-yes, 0-no) |
| SCQ_total_scores | Total scores in Social Communication Questionnaire |
| ADOS_Repetetive_behaviour | The algorithm value in repetitive behavior in ADOS-2 |
| ADOS_Communication | The algorithm value in communication in ADOS-2 |
| ADOS_Social | The algorithm value in social functioning in ADOS-2 |
| ADOS_mod_3cutoff | Cut off scores in module 3 in ADOS- 2 (1-yes, 0-no) |
| ADOS_mod_4cutoff | Cut off scores in module 4 in ADOS- 2 (1-yes, 0-no) |
| ADI-R_social | The algorithm value in social functioning in ADI-R |
| ADI-R_communication | The algorithm value in social functioning in communication in ADI-R |
| ADI-R_repetetive_behaviour | The algorithm value inrepetive and restricted behaviour in ADI-R |
