# Supplementary material for: Efficiency of attentional processes in attention network theory and autistic symptoms in adolescents with autism spectrum disorder
Source: Front Psychiatry. 2022 Oct 12;13:950245. doi: 10.3389/fpsyt.2022.950245 (PMC9614655; doi:10.3389/fpsyt.2022.950245)
Supplement: Supplementary file 3 [file Table_2.DOCX]

**Supplementary Material**

The Kolmogorow- Smirnow statistics in ASD group and control group

|  | ASD group (N=37) | | Control group (N=37) | |
| --- | --- | --- | --- | --- |
| Attention Network Test (ANT) | Statistic | p-value (2- sided) | Statistic | p-value |
| alerting | 0,102 | 0,2 | 0,09 | 0,2 |
| RT no cue condtion | 0,152 | 0,03 | 0,124 | 0,163 |
| RT with double cue | 0,142 | 0,057 | 0,114 | 0,2 |
| Correct responses with no cue | 0,173 | 0,007 | 0,329 | 0,001 |
| Correct responses with double cue | 0,162 | 0,016 | 0,307 | 0,001 |
| orienting | 0,143 | 0,054 | 0,145 | 0,047 |
| RT center cue | 0,123 | 0,173 | 0,127 | 0,139 |
| RT spatial cue | 0,100 | 0,200 | 0,123 | 0,173 |
| Correct responses with center cue | 0,145 | 0,046 | 0,319 | 0,001 |
| Correct responseswith spatial cue | 0,162 | 0,015 | 0,387 | 0,001 |
| executive attention | 0,173 | 0,007 | 0,191 | 0,002 |
| RT congruent flanker | 0,123 | 0,168 | 0,198 | 0,001 |
| RT incongruent flanker | 0,142 | 0,058 | 0,127 | 0,136 |
| RT neutral flanker | 0,123 | 0,169 | 0,176 | 0,005 |
| Correct responses congruent flanker | 0,163 | 0,014 | 0,381 | 0,001 |
| Correct responses incongruent flanker | 0,138 | 0,072 | 0,373 | 0,001 |
| Correct responses neutral flanker | 0,213 | 0,001 | 0,332 | 0,001 |
| Color Trails Test (CTT) |  |  |  |  |
| performing time of CTT-1 | 0,206 | 0,001 | 0,12 | 0,2 |
| performing time of CTT-2 | 0,211 | 0,001 | 0,151 | 0,033 |
| intereference index CTT | 0,141 | 0,061 | 0,116 | 0,2 |
| Test of concentration d2 |  |  |  |  |
| d2 test- perecentage of errors | 0,237 | 0,001 | 0,278 | 0,001 |
| d2Concentration performance (CONC) | 0,095 | 0,200 | 0,137 | 0,076 |
| Wisconsin Card Sorting Test (WCST) |  |  |  |  |
| the number of correct responsesWCST | 0,225 | 0,001 | 0,151 | 0,033 |
| WCSTthe percentage of perseverative responses and errors | 0,268 | 0,001 | 0,168 | 0,01 |

RT- reaction time
